# Incremental Anatomical Value of CT Angiography Beyond Echocardiography in the Preoperative Assessment of Pediatric Conotruncal Anomalies: A Surgical Correlation Study

**DOI:** 10.3390/diagnostics16193105

**Published:** 2026-09-24

**Authors:** Demet Kangel, Burcu Çevlik, Elnur Karimov, Ahmet Saki Oğuz, Selin Sağlam, Behzat Tüzün, Ali Can Hatemi, Serap Baş, Erkut Öztürk

**Affiliations:** 1 Department of Pediatric Cardiology, University of Health Sciences, Basaksehir Cam and Sakura City Hospital, Istanbul 34480, Turkey; drburcudrl@gmail.com (B.Ç.); elnurkarimov91@gmail.com (E.K.); ahmetsaki@gmail.com (A.S.O.); erkut.ozturk@sbu.edu.tr (E.Ö.); 2 Department of Anaesthesiology and Reanimation, University of Health Sciences, Basaksehir Cam and Sakura City Hospital, Istanbul 34480, Turkey; selinsaglams@gmail.com; 3 Department of Pediatric Cardiovascular Surgery, University of Health Sciences, Basaksehir Cam and Sakura City Hospital, Istanbul 34480, Turkey; bavebeyza@hotmail.com (B.T.); alican.hatemi@sbu.edu.tr (A.C.H.); 4 Department of Radiology, University of Health Sciences, Basaksehir Cam and Sakura City Hospital, Istanbul 34480, Turkey

**Keywords:** conotruncal anomalies, computed tomography angiography, echocardiography, congenital heart disease, preoperative imaging, surgical planning

## Abstract

**Background:** Conotruncal anomalies frequently involve extracardiac vascular structures that are critical for surgical planning yet incompletely assessed by transthoracic echocardiography (TTE). We evaluated the incremental diagnostic value of CT angiography (CTA) beyond TTE using intraoperative findings as the reference standard. **Methods:** In this single-center retrospective study, 239 children who underwent surgical repair of a conotruncal anomaly and had both preoperative TTE and CTA were analyzed. The incremental contribution of CTA was graded as none, minor, or major by two independent reviewers. Diagnostic performance of each modality was compared with operative findings for coronary artery anomalies, right aortic arch, aortic arch hypoplasia, interrupted aortic arch, and pulmonary artery atresia, non-confluence, and hypoplasia. **Results:** CTA provided incremental information beyond TTE in 232 patients (97.1%), classified as major in 51.9% and minor in 45.2%; interobserver agreement was almost perfect (quadratic-weighted κ = 0.825). CTA was superior for coronary artery anomalies (sensitivity 91.7% vs. 50.0%), branch pulmonary artery hypoplasia (97.3% vs. 89.3%), and aortic arch hypoplasia (100% vs. 87.5%; all *p* < 0.05), and identified aortopulmonary collaterals and a persistent left superior vena cava more frequently than TTE. The two modalities performed comparably for aortic arch sidedness and interrupted aortic arch, and pulmonary atresia. **Conclusions:** CTA provides additional, operatively confirmed anatomical information beyond TTE in the majority of children with conotruncal anomalies, with the greatest value for extracardiac vascular anatomy. Rather than replacing echocardiography, CTA should be regarded as a complementary modality that refines preoperative anatomical assessment.

## 1. Introduction

Conotruncal anomalies (CTAs), including tetralogy of Fallot (TOF), transposition of the great arteries (TGA), double-outlet right ventricle (DORV), pulmonary atresia with ventricular septal defect (PA-VSD), truncus arteriosus, interrupted aortic arch (IAA), and aortopulmonary window (APW), arise from abnormal neural crest cell migration and second heart field development during cardiac embryogenesis [1,2,3]. With a prevalence of approximately 11.6 per 10,000 live births and accounting for 10–30% of critical congenital heart disease, CTAs represent a major group of congenital cardiac malformations in children [4,5]. These lesions are frequently associated with genetic abnormalities, particularly 22q11.2 deletion syndrome, increasing perioperative complexity and morbidity [5,6]. Despite substantial advances in neonatal and infant cardiac surgery, accurate preoperative assessment of great vessel anatomy, coronary arteries, pulmonary arteries, aortic arch abnormalities, and associated vascular anomalies remains crucial for surgical planning and outcomes [3,4,5,6,7].

Transthoracic echocardiography (TTE) remains the cornerstone of the initial evaluation of conotruncal anomalies, providing real-time, radiation-free assessment of intracardiac anatomy, ventricular function, and hemodynamics [8,9]. Its portability and widespread availability make it indispensable during the neonatal period, when acoustic windows are generally favorable [8]. However, TTE is operator-dependent and limited by acoustic window constraints, particularly in patients with lung hyperinflation, chest wall deformities, or previous thoracic surgery [8,10]. In addition, extracardiac vascular structures may be incompletely visualized, including coronary artery anatomy, distal pulmonary arteries, aortic arch branching patterns, major aortopulmonary collateral arteries (MAPCAs), and systemic venous anomalies [7,11]. In a large retrospective surgical correlation study, approximately 27% of pediatric patients with congenital heart disease required additional imaging before surgery, most commonly for assessment of coronary anatomy, pulmonary artery morphology, and associated vascular abnormalities [12]. These limitations are particularly relevant in conotruncal anomalies, where unrecognized extracardiac vascular anatomy may directly affect surgical planning and outcomes.

The advent of multidetector computed tomography (MDCT) has transformed preoperative imaging in conotruncal anomalies. Modern CT systems provide submillimeter spatial resolution with rapid acquisition times, reducing motion artifacts and the need for sedation in neonates and infants [13,14]. Prospective ECG-gated techniques enable reliable assessment of coronary artery anatomy while maintaining low radiation exposure [14,15]. In addition, volumetric datasets allow comprehensive multiplanar and three-dimensional reconstructions, providing accurate visualization of thoracic cardiovascular anatomy in a single non-invasive examination [3,11,13]. Compared with TTE, CTA offers superior evaluation of extracardiac vascular anatomy, including aortic arch abnormalities, distal pulmonary arteries, coronary artery anomalies, MAPCAs, and systemic venous anomalies, all of which are highly relevant for surgical planning [3,16,17].

Despite increasing evidence supporting CTA in selected conotruncal anomalies, most studies remain disease-specific, focusing primarily on TOF [16,18], TGA [19], or PA-VSD and MAPCAs [20]. Consequently, studies evaluating the broader conotruncal spectrum and directly comparing TTE and CTA with surgical correlation are limited [3,17]. Furthermore, the incremental diagnostic value of CTA beyond TTE has not been systematically assessed using a standardized framework across major anatomical domains relevant to surgical planning [3,13]. Previous surgical correlation studies have also been limited by small sample sizes, heterogeneous populations, and inadequate evaluation of clinically relevant discordant findings between imaging modalities [15,18]. As a result, the extent and anatomical distribution of the additional diagnostic information provided by CTA remain incompletely defined for the conotruncal spectrum as a whole.

Specifically, we sought to quantify its incremental diagnostic value, compare the domain-specific diagnostic performance of the two modalities against surgical findings, and identify the anatomical domains in which CTA provides the greatest additional information for preoperative planning.

## 2. Methods

### 2.1. Study Design & Patient Selection

This retrospective observational study was conducted at a tertiary care center where pediatric congenital cardiac surgery is performed. The study protocol was approved by the institutional ethics committee (approval number: 354; date: 26 November 2025). Study-specific informed consent was waived owing to the retrospective design and the use of fully anonymized data. In addition, written general consent for the use of anonymized clinical records and imaging data for scientific research had been obtained from the legal guardians of all patients at the time of hospital admission. All CTA examinations included in this study had been performed for standard clinical indications as part of routine preoperative surgical planning; no imaging, contrast administration, or sedation was undertaken for research purposes, and the ethics approval therefore concerned the retrospective analysis of existing clinical data. Consecutive patients who underwent primary cardiac surgery for a conotruncal anomaly between 1 September 2023 and 1 September 2025 were screened.

Eligible patients were younger than 18 years, had a confirmed conotruncal anomaly, underwent both preoperative TTE and CTA, and had operative records available for surgical correlation. Conotruncal anomalies included TOF, PA-VSD, TGA, DORV, truncus arteriosus, IAA, and APW.

At our institution, preoperative CTA is performed as part of a standardized protocol for anatomical assessment across the full spectrum of conotruncal anomalies—including TOF, PA-VSD, TGA, DORV, truncus arteriosus, IAA, APW—in which detailed delineation of the coronary arteries, pulmonary arteries, aortic arch, and extracardiac vascular anatomy is routinely required for surgical planning. CTA was therefore obtained on the basis of the underlying diagnosis rather than being triggered by specific echocardiographic findings or diagnostic uncertainty. This diagnosis-based protocol was already established as part of routine institutional practice before the study period and was applied irrespective of echocardiographic image quality. Accordingly, the study cohort represents consecutive surgical patients across these diagnostic groups in whom both TTE and CTA were available, rather than a population selected because echocardiography was inconclusive.

Patients were excluded if they had: (1) complex associated cardiac anatomy likely to confound imaging analysis, such as heterotaxy/isomerism or complete atrioventricular septal defect; (2) absence of surgical treatment or missing operative records precluding surgical correlation; (3) contraindication to iodinated contrast administration or renal dysfunction precluding CTA; (4) inadequate CTA image quality for diagnostic interpretation; or (5) previous palliative surgical intervention before CTA, including systemic-to-pulmonary shunt or pulmonary artery banding.

A total of 239 patients met the eligibility criteria and were included in the final analysis.

### 2.2. Imaging Protocols

#### 2.2.1. CT Angiography

All CT examinations were performed using a 640-slice multidetector CT system (Aquilion ONE, GENESIS Edition; Canon Medical Systems, Otawara, Japan). ECG-gated volumetric acquisitions were obtained in axial mode with a gantry rotation time of 0.35 s. Tube voltage was adjusted according to body weight, with 80 kV used in patients weighing less than 35 kg, while tube current was regulated using automated exposure control. Intravenous iodinated contrast material (300 mgI/mL) was administered at 1.5 mL/kg body weight, followed by a saline flush. All examinations were performed during free breathing. The majority of CTA examinations were performed without sedation using a feed-and-wrap technique, facilitated by the rapid single-heartbeat acquisition; when sedation was clinically required, it was administered by a cardiac anesthesiologist. Images were reconstructed with a slice thickness of 0.5 mm using an iterative reconstruction algorithm. Post-processing included multiplanar reformation (MPR), maximum intensity projection (MIP), and three-dimensional volume-rendered (VR) reconstructions. All CTA datasets were reviewed by a pediatric cardiovascular radiologist with at least 15 years of experience in congenital heart disease imaging.

A low-dose prospective ECG-gated protocol with weight-adapted tube voltage and automated tube-current modulation was used for all examinations. For each examination, the dose–length product (DLP) was recorded. The DLP and conversion factors were referenced to a 32 cm body phantom; therefore, the DLP was multiplied by two to adjust to the 16 cm phantom reference used for pediatric thoracic dosimetry. The effective dose (ED) was then estimated as the product of the DLP and an age-specific thoracic conversion coefficient (k), using 0.039 mSv·mGy^−1^·cm^−1^ for neonates and infants, 0.026 for children aged 1–5 years, 0.018 for those aged 5–10 years, and 0.014 for those older than 10 years [21].

#### 2.2.2. Transthoracic Echocardiography

All TTE examinations were performed using the Philips Affiniti 50 ultrasound system (Philips, Bothell, WA, USA) with 9 and 12 MHz transducers. Studies were conducted by pediatric cardiologists with more than 10 years of experience in congenital heart disease imaging, in accordance with the guidelines of the American Society of Echocardiography. Standard pediatric views were obtained, including parasternal long- and short-axis, apical four- and five-chamber, subcostal, and suprasternal projections. Cardiac morphology was evaluated using a segmental approach encompassing atrial situs, venoatrial connections, atrioventricular and ventriculoarterial connections, great artery spatial relationships, intracardiac defects, coronary artery origins, and extracardiac vascular anomalies. Two-dimensional, M-mode, color Doppler, pulsed-wave Doppler, and continuous-wave Doppler interrogation were applied systematically. No sedation was administered during TTE examinations.

### 2.3. Data Collection

Demographic and clinical data included age at CTA, sex, body weight, and body surface area (BSA; calculated using the Mosteller formula). Weight, height, and body mass index were expressed as percentiles and standard deviation scores (SDS) derived from World Health Organisation (WHO) and Centers for Disease Control and Prevention (CDC) growth references using the LMS (lambda–mu–sigma) method, according to age and sex. Imaging-related parameters included preoperative oxygen saturation, contrast volume administered during CTA, and the estimated effective radiation dose. For both TTE and CTA, the following anatomical domains were systematically evaluated and recorded as present or absent: coronary artery origin and course; pulmonary artery morphology (confluence, size, stenosis, or atresia); aortic arch anatomy (sidedness, branching pattern, hypoplasia, or interruption); aortopulmonary collateral arteries (MAPCAs); systemic venous anomalies, including persistent left superior vena cava (PLSVC); and pulmonary venous return anomalies. Surgical data included the type of repair performed and intraoperative anatomical findings documented in the operative notes, which served as the reference standard for diagnostic correlation. Operative findings were extracted retrospectively from surgical records. As these records were generated by surgeons who had access to the preoperative CTA, the reference standard was not fully independent of the index test for structures routinely visualized during surgery. Furthermore, because certain extracardiac structures—particularly aortopulmonary collaterals and the drainage of systemic venous anomalies—are not systematically explored or documented during conotruncal repair, the absence of such a finding in the operative record could not be reliably equated with its true anatomical absence. Formal diagnostic performance was therefore assessed only for domains routinely confirmed at surgery (coronary artery anomalies, right aortic arch, aortic arch hypoplasia, and IAA), whereas MAPCAs and PLSVC were reported descriptively.

### 2.4. Outcome Definitions

The primary outcome was the incremental diagnostic contribution of CTA beyond TTE, categorized using a three-tier scoring system: 0 = no additional information (CTA findings were concordant with TTE, with no additional anatomical information provided); 1 = minor additional anatomical information (CTA identified additional anatomical findings or clarified anatomical details incompletely characterized by TTE); and 2 = major additional anatomical information (CTA provided anatomically significant information, particularly regarding coronary arteries, aortopulmonary collaterals, pulmonary artery morphology, aortic arch anatomy, or systemic/pulmonary venous anomalies, that was incompletely characterized by TTE). Scores were assigned using these predefined, rule-based criteria rather than a subjective impression of importance. Because the major category required correlation with operative findings, reviewers necessarily had access to the operative records; scoring was therefore based on predefined anatomical domains and operative confirmation rather than on a retrospective impression of clinical relevance. TTE and CTA were assessed independently by two experienced pediatric cardiologists, with each modality scored separately. Interobserver agreement was assessed using the quadratic-weighted Cohen’s kappa coefficient, and discordant cases were resolved by consensus.

Secondary outcomes included domain-specific diagnostic performance of TTE and CTA compared with intraoperative findings, which served as the reference standard. Formal diagnostic performance analyses were performed for coronary artery anomalies, right-sided aortic arch, aortic arch hypoplasia, and IAA, and pulmonary artery atresia, non-confluence, and hypoplasia. MAPCAs and PLSVC were reported descriptively, because the operative record does not provide a reliable reference standard for structures that are not systematically explored during surgery. Sensitivity, specificity, positive predictive value (PPV), negative predictive value (NPV), and overall diagnostic accuracy were calculated independently for both modalities. Pulmonary venous return anomalies were reported descriptively because of the limited number of positive cases.

### 2.5. Statistical Analysis

Statistical analyses were performed using SPSS Statistics version 25.0 (IBM Corporation, Armonk, NY, USA). Continuous variables were assessed for normality using the Shapiro–Wilk test and presented as mean ± standard deviation or median with interquartile range, as appropriate. Categorical variables were expressed as frequencies and percentages.

For the primary outcome, the distribution of scores (0 = no, 1 = minor, and 2 = major additional anatomical information) was analyzed descriptively across the study cohort and diagnostic subgroups. Interobserver agreement for score assignment was assessed using the quadratic-weighted Cohen’s kappa coefficient, with 95% confidence intervals estimated by bootstrapping (2000 resamples). Associations between incremental contribution category and conotruncal anomaly subtype were evaluated using the Freeman–Halton extension of Fisher’s exact test, given the small expected cell counts in several subgroups. For secondary outcomes, sensitivity, specificity, positive predictive value (PPV), negative predictive value (NPV), and overall diagnostic accuracy were calculated independently for TTE and CTA in each anatomical domain, with intraoperative findings serving as the reference standard. Paired proportions between imaging modalities were compared using the McNemar test, and agreement between imaging findings and surgical findings was assessed using Cohen’s kappa coefficient. Given the multiple anatomical domains and diagnostic metrics examined, the domain-specific comparisons were considered exploratory, and *p*-values were not adjusted for multiplicity; emphasis was therefore placed on effect sizes and 95% confidence intervals rather than on significance thresholds, and comparisons based on small numbers of positive or discordant cases were interpreted with caution.

A two-tailed *p* value < 0.05 was considered statistically significant for all analyses.

## 3. Results

During the study period, 296 patients underwent surgery for a conotruncal anomaly, of whom 239 met the eligibility criteria and were included in the final analysis (Figure 1). Of the 57 excluded patients, 23 had undergone prior palliative surgery, 21 lacked preoperative CTA (17 for clinical or logistic reasons and 4 owing to renal dysfunction precluding contrast administration), and 13 had missing operative records precluding surgical correlation. Of the 239 included patients, 150 (62.8%) were male. Median age at CTA was 120 days (IQR 4–196), with 215 patients (90.0%) imaged during the neonatal or infant period, and CTA preceded surgery by a median of 7 days (IQR 4–13). TTE preceded CTA by a median of 1 day (IQR 1–2; range 0–5). Median weight, height, and BMI standard deviation scores were −0.84, −0.91, and −0.56, respectively. The most frequent diagnosis was TOF (94, 39.3%), followed by DORV (50, 20.9%) and TGA (44, 18.4%); the remaining diagnoses were PA-VSD, truncus arteriosus, aortopulmonary window, and IAA. Median preoperative oxygen saturation was 89% (IQR 83–95). CTA was performed with a median contrast volume of 1.4 mL/kg (IQR 1.2–1.6) and a median estimated effective dose of 2.18 mSv (IQR 1.79–2.82). Baseline characteristics are summarized in Table 1.

When the added value of CTA over TTE was graded for each patient, an incremental contribution was recorded in 232 of 239 cases (97.1%). This contribution was classified as major additional anatomical information in 124 patients (51.9%) and minor additional anatomical information in 108 (45.2%), while no additional information was gained in only 7 patients (2.9%). The proportion classified as major additional anatomical information varied across diagnostic subgroups, from 42.6% in TOF to 76.9% in PA-VSD, although this difference did not reach statistical significance (Freeman–Halton extension of Fisher’s exact test, *p* = 0.057). The distribution of contribution scores by diagnosis is shown in Table 2. Agreement between the two independent reviewers who assigned these scores was almost perfect (quadratic-weighted κ = 0.825, 95% CI 0.763–0.881; raw agreement 214/239, 89.5%; Supplementary Table S1), and every discordant case differed by only a single category.

The diagnostic performance of each modality against intraoperative findings is detailed in Table 3, and the corresponding contingency data with paired comparisons are in Table 4. For the identification of coronary artery anomalies, TTE performed poorly, with a sensitivity of 50.0% and only fair agreement with surgical findings (κ = 0.421); CTA raised the sensitivity to 91.7% and yielded almost perfect agreement (κ = 0.932), a difference that was highly significant (McNemar *p* < 0.001). A comparable advantage was seen for aortic arch hypoplasia, where CTA correctly classified all 24 surgically confirmed cases (sensitivity 100%, κ = 1.000) compared with a sensitivity of 87.5% for TTE (κ = 0.841, *p* = 0.016).

Aortopulmonary collateral arteries and systemic venous anomalies were assessed descriptively, as these structures are not systematically explored at surgery and therefore lack a reliable operative reference standard. Major aortopulmonary collateral arteries were surgically confirmed in 47 patients; CTA identified 44 of these (94%) and TTE 30 (64%), consistent with the recognized difficulty of delineating collateral vessels by echocardiography. A persistent left superior vena cava was present in 17 patients; CTA detected all 17, whereas TTE identified 11, with the remaining cases recognized on CTA through direct visualization of the anomalous vessel and its drainage.

For the remaining two domains, the two modalities performed similarly. TTE reliably established the side of the aortic arch (sensitivity 87.5%, κ = 0.892), and although CTA again showed marginally higher concordance (κ = 0.942), the difference was not significant (*p* = 0.388). IAA was likewise identified by both modalities with high accuracy and without a statistically significant difference (TTE κ = 0.880 versus CTA κ = 1.000; *p* = 0.125).

Pulmonary artery morphology was systematically assessed in all patients. At surgery, pulmonary artery hypoplasia was confirmed in 112 patients (46.9%), pulmonary atresia in 25 (10.5%), and non-confluent pulmonary arteries in 8 (3.3%). CTA delineated the central pulmonary arteries in detail—confluence, branching pattern, calibre, and the site and extent of hypoplasia or atresia—and in patients with pulmonary atresia or diminutive arteries it clarified the source of pulmonary blood supply and the continuity of the branch pulmonary arteries, anatomical features that are central to operative planning yet difficult to capture fully on echocardiography. Pulmonary artery atresia, non-confluence, and hypoplasia were included in the formal comparison against surgical findings. CTA showed higher sensitivity than TTE for all three: pulmonary atresia (96.0% vs. 92.0%; CTA κ = 0.955), non-confluence (87.5% vs. 50.0%; CTA κ = 0.931), and branch hypoplasia (97.3% vs. 89.3%; CTA κ = 0.975), with the largest advantage for branch hypoplasia (McNemar *p* = 0.022) and non-confluence. These findings are consistent with the recognized limitation of echocardiography in delineating the branch and distal pulmonary arteries.

Anomalies of pulmonary venous return were infrequent. No patient had a total anomalous pulmonary venous connection, and a partial anomalous connection was present in a single patient, which was detected by CTA and confirmed at surgery but overlooked on echocardiography. Because of the small number of positive cases, this domain was analyzed descriptively only and was not subjected to formal performance testing.

## 4. Discussion

In this retrospective cohort of children with conotruncal anomalies undergoing surgical repair, CTA provided additional diagnostic information beyond TTE in a substantial proportion of patients, with intraoperative findings serving as the reference standard. The incremental value of CTA was greatest for extracardiac vascular structures that are often incompletely visualized by TTE, particularly coronary artery anatomy, MAPCAs, PLSVC, and aortic arch hypoplasia, whereas both modalities showed comparable performance for anatomical features that lie well within the echocardiographic window, such as aortic arch sidedness and IAA. This domain-specific pattern, rather than a uniform superiority of one modality, underscores the role of CTA in defining vascular anatomy that is incompletely characterized by TTE alone. Together, these findings support the complementary use of CTA in the preoperative evaluation of conotruncal anomalies when detailed extracardiac anatomical assessment is required for surgical planning.

The most striking difference between the two modalities was observed for coronary artery anomalies, where TTE detected fewer than half of the surgically confirmed cases (sensitivity 50%), whereas CTA identified 91.7% of cases, in near-perfect agreement with operative findings. This discrepancy reflects the well-recognized limitations of TTE in delineating coronary origins and proximal course, particularly in neonates and small infants with limited acoustic windows. Accurate preoperative characterization of coronary anatomy is critical throughout the conotruncal spectrum because coronary variants may substantially influence surgical strategy. In TOF, a coronary artery crossing the right ventricular outflow tract may preclude a transannular incision and necessitate an alternative repair strategy [22], whereas in TGA and Taussig–Bing physiology, coronary anatomy directly affects the complexity and risk of coronary transfer during arterial switch procedures [7,23]. Previous studies have demonstrated that CTA depicts coronary anatomy with accuracy approaching that of invasive angiography [16,24], and our findings extend this evidence by demonstrating superior detection of coronary anomalies compared with echocardiography using surgical findings as the reference standard across a broad spectrum of conotruncal anomalies. As an illustrative example, CTA identified an anomalous left circumflex coronary artery arising from the right coronary artery in a patient with d-TGA (Figure 2).

A similar pattern was observed for aortopulmonary collateral vessels, which CTA detected more frequently than TTE (44 of 47 vs. 30 of 47 surgically confirmed cases). These vessels arise from the descending aorta or its branches and often follow a complex and variable course that is difficult to delineate by TTE, whereas CTA can define their origin, number, distribution, connection and relationship to the native pulmonary arteries in a single examination [25]. Such information is potentially relevant to surgical planning across the conotruncal spectrum, particularly in PA-VSD, where pulmonary blood flow may depend heavily on collateral supply, and in TOF, where unrecognized collaterals may contribute to pulmonary overcirculation, excessive surgical bleeding, and adverse perioperative outcomes [16,26]. Although collateral detection does not necessarily alter the primary diagnosis, it frequently provides important anatomical information that may influence operative planning and perioperative management.

The detection of PLSVC further illustrated the complementary value of the two modalities. CTA identified all 17 surgically confirmed cases, whereas TTE identified 11. Although PLSVC is often clinically silent, its preoperative recognition is important in conotruncal surgery because an unanticipated left-sided superior vena cava may complicate venous cannulation, retrograde cardioplegia delivery, and cavopulmonary procedures requiring balanced systemic venous drainage [27]. On echocardiography, a dilated coronary sinus may suggest the diagnosis; however, the full course and drainage pattern of the anomalous vessel are not always adequately visualized [28]. In our cohort, the lower echocardiographic sensitivity for PLSVC likely also reflects its retrospective ascertainment, as systemic venous anomalies were not always explicitly documented in the original echocardiographic reports; the magnitude of this difference may therefore be institution-dependent. In contrast, CTA provides comprehensive visualization of the systemic venous anatomy and reliably demonstrates both the course and termination of the vessel [29]. Our findings therefore suggest that CTA may be particularly valuable when systemic venous anatomy remains uncertain after echocardiographic assessment. As an illustrative example, CTA demonstrated a PLSVC draining into the coronary sinus in a patient with DORV (Figure 3).

In contrast to the extracardiac vascular domains discussed above, TTE and CTA showed comparable performance for several aspects of aortic arch anatomy. TTE reliably identified arch sidedness (sensitivity 87.5%) and interrupted aortic arch, with no significant difference compared with CTA. This finding is not unexpected, as the aortic arch and its major branches are generally well visualized from the suprasternal echocardiographic window and have long been considered strengths of echocardiographic assessment [30]. CTA nevertheless retained an advantage for aortic arch hypoplasia, demonstrating perfect agreement with surgical findings (κ = 1.000), whereas TTE was somewhat less sensitive. This likely reflects the ability of cross-sectional imaging to provide more reproducible assessment of arch dimensions and the extent of narrowing [31,32]. Thus, while echocardiography is usually sufficient for determining arch sidedness and detecting interruption, CTA offers complementary information that may improve characterization of complex arch anatomy relevant to surgical planning. For example, CTA precisely delineated a type B interrupted aortic arch, defining the site and length of the interrupted segment (Figure 4), and demonstrated hypoplasia of the aortic arch and isthmus in a neonate with a Taussig–Bing anomaly (Figure 5).

Beyond its performance in individual anatomical domains, a notable finding of this study was the near-universal diagnostic yield of CTA across the conotruncal spectrum. CTA provided incremental information beyond TTE in 97% of patients and failed to add value in only 3%, indicating that its contribution was not confined to a limited number of lesion types but extended consistently across diagnostic subgroups. Although the proportion of major additional anatomical information appeared to vary between subgroups, ranging from 42.6% in TOF to over 75% in PA-VSD and APW, this difference did not reach statistical significance (*p* = 0.057), and the relatively small number of patients in some subgroups precludes firm conclusions regarding lesion-specific differences. Importantly, findings classified as minor additional anatomical information were not clinically trivial: identification of a persistent left superior vena cava, an aortopulmonary collateral vessel, or the precise extent of aortic arch hypoplasia may not alter the primary diagnosis but can meaningfully influence operative planning and perioperative management [26,27,33]. Taken together, these findings suggest that CTA provides clinically relevant information in the vast majority of patients with conotruncal anomalies and may serve as a valuable complement to echocardiography during preoperative assessment.

A recognized limitation of CTA compared with TTE is exposure to ionizing radiation, an important consideration in neonates and infants. However, this concern must be balanced against the diagnostic benefit provided by CTA and the radiation burden of alternative investigations, particularly diagnostic cardiac catheterization. Advances in CT technology and dose-reduction strategies have substantially lowered radiation exposure in pediatric cardiovascular imaging, allowing high-quality diagnostic studies to be performed at relatively low doses [34]. Our protocol incorporated contemporary low-dose principles, including prospective ECG-gated acquisition and weight-adapted imaging parameters, in accordance with current radiation safety recommendations [35]. In our cohort, the median estimated effective dose was 2.18 mSv (IQR 1.79–2.82), consistent with contemporary low-dose pediatric cardiac CT protocols and well below the exposure associated with diagnostic cardiac catheterization. Within this context, the substantial incremental diagnostic value observed in the present study suggests that CTA can provide clinically important information at an acceptable radiation cost in children with conotruncal anomalies. In these diagnostic groups, preoperative delineation of the coronary arteries, pulmonary arteries, and aortic arch is essential for surgical planning and is supported by conotruncal- and lesion-specific evidence as well as expert consensus recommendations for cardiovascular CT in congenital heart disease [3,10,17,35]; CTA was therefore performed as part of standard clinical care rather than as an additional research procedure, and no incremental radiation or contrast exposure was incurred for the purposes of this study.

## 5. Limitations

This study has several limitations. First, it was a single-center retrospective analysis, which may introduce selection bias and limit the generalizability of the findings. Second, although the overall cohort was relatively large, several diagnostic subgroups—particularly truncus arteriosus, APW, and IAA—contained small numbers of patients, limiting the statistical power for lesion-specific analyses and likely contributing to the borderline significance observed in subgroup comparisons. Third, pulmonary venous return anomalies were analyzed descriptively rather than through formal diagnostic performance metrics because of the small number of positive cases. Fourth, because operative records were generated with knowledge of the CTA findings, the reference standard was not fully independent of the index test; this incorporation bias may have favored the more detailed modality (CTA) and could have led to an overestimation of its diagnostic performance. Fifth, the incremental value of CTA was assessed retrospectively from imaging and operative records rather than through a prospective comparison of surgical plans before and after CTA; accordingly, the major category denotes a finding that provided substantial additional anatomical information confirmed at surgery, rather than direct evidence that CTA changed surgical management. Preoperative imaging pathways may differ across institutions, and our diagnosis-based CTA protocol reflects local practice rather than a universal standard; centers with different echocardiographic capabilities or imaging protocols may adopt more selective indications for CTA. Prospective multicenter studies with standardized imaging protocols are warranted to validate and extend these findings.

## 6. Conclusions

In this cohort of children with conotruncal anomalies, CTA provided incremental diagnostic information beyond TTE in the vast majority of patients, with the greatest added value for extracardiac vascular structures such as the coronary arteries, MAPCAs, PLSVC, and aortic arch hypoplasia. For anatomical features that lie well within the echocardiographic window, including arch sidedness and IAA, the two modalities performed comparably. Rather than replacing TTE, CTA is best regarded as a complementary modality that, when integrated into preoperative assessment, provides additional, operatively confirmed anatomical information that may influence surgical planning and perioperative management across the conotruncal spectrum.

## Figures and Tables

**Figure 1:**
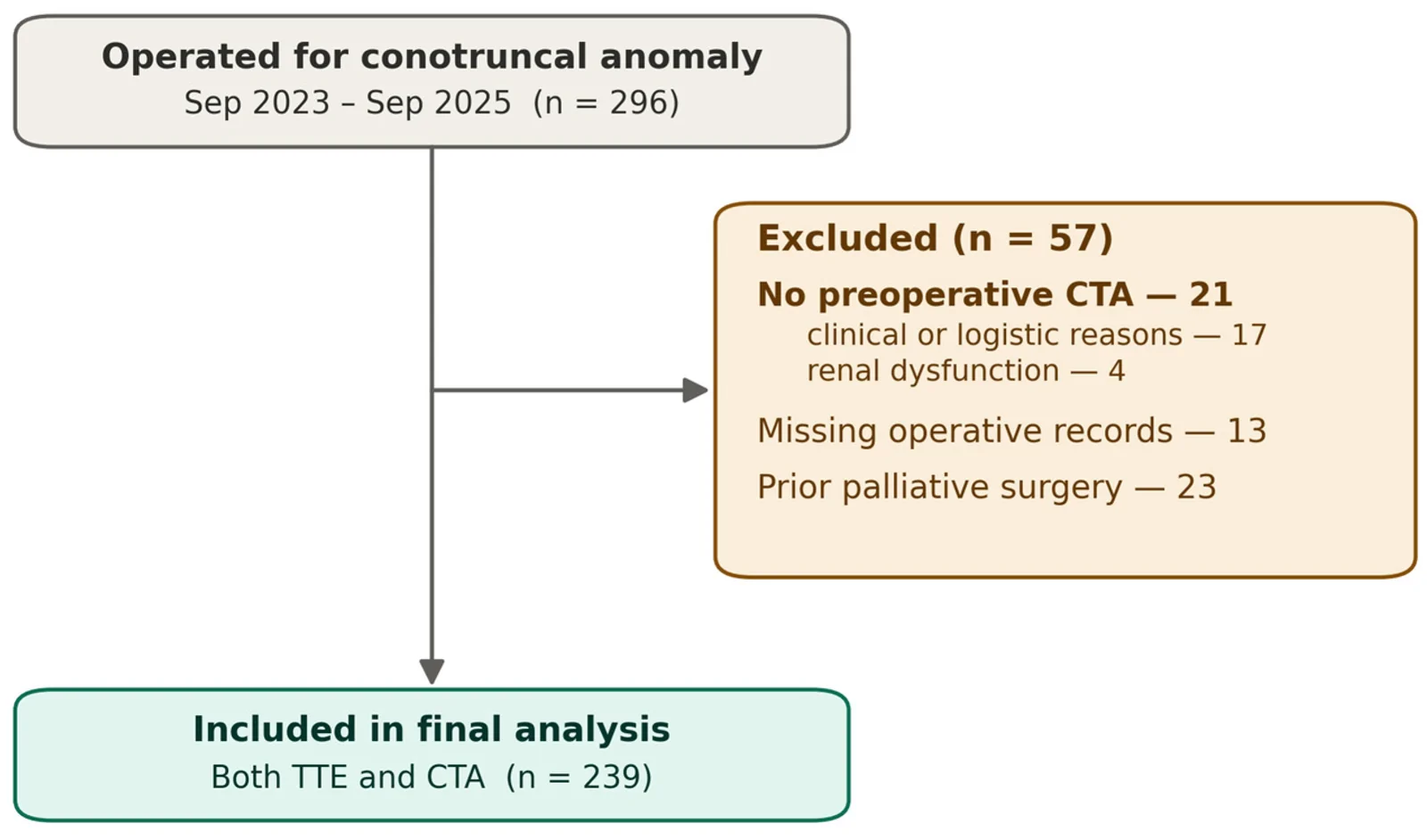
Study flow diagram. CTA, computed tomography angiography; TTE, transthoracic echocardiography.

**Figure 2:**
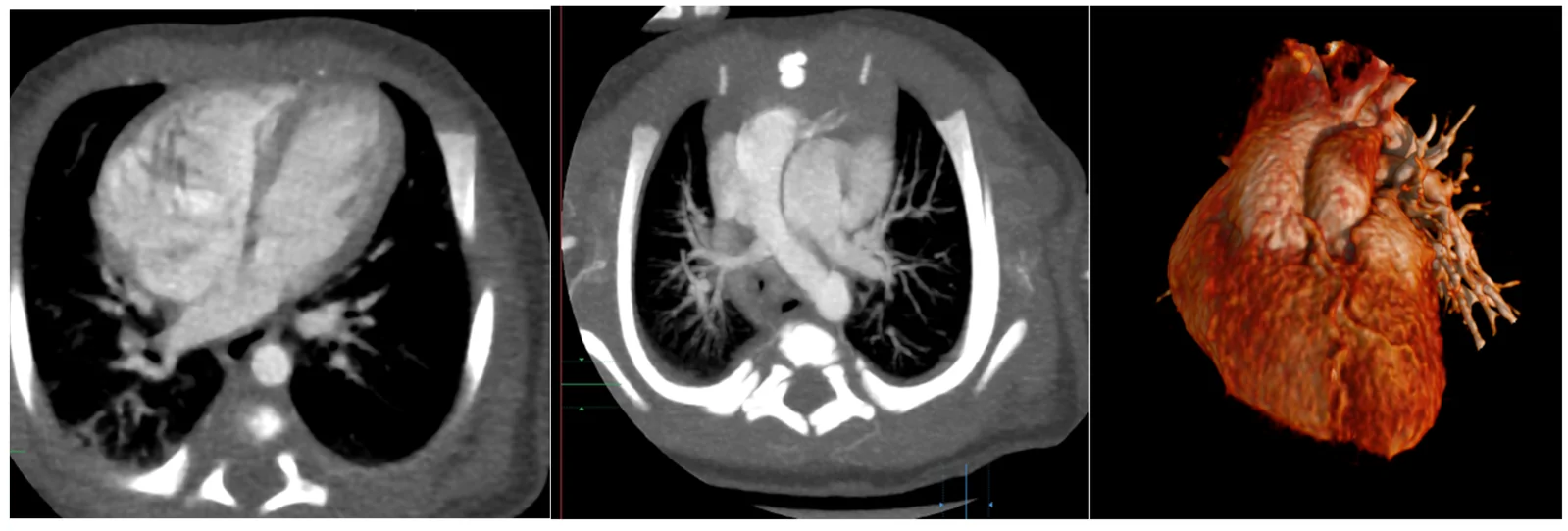
A patient with dextro-transposition of the great arteries (d-TGA) and an anomalous left circumflex coronary artery arising from the right coronary artery.

**Figure 3:**
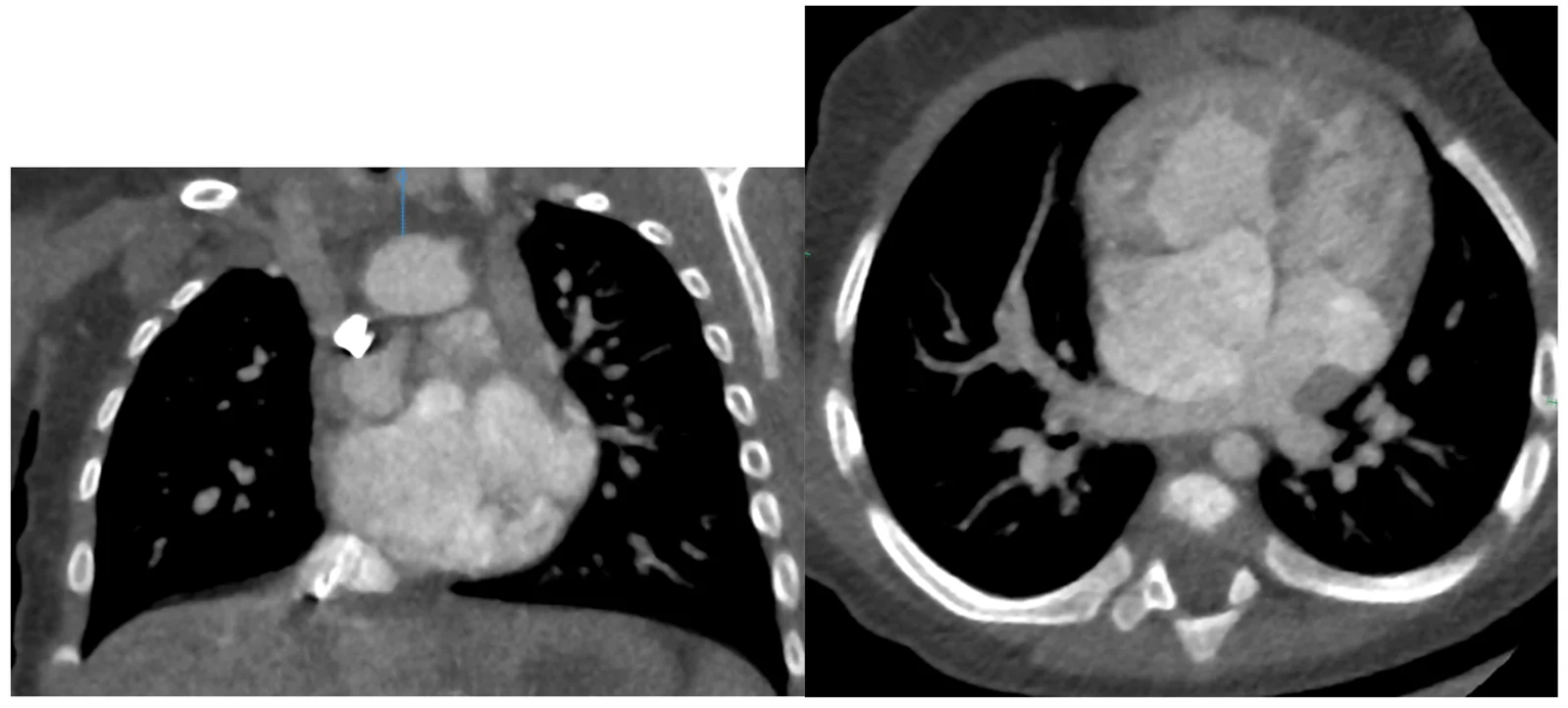
A patient with a persistent left superior vena cava (PLSVC).

**Figure 4:**
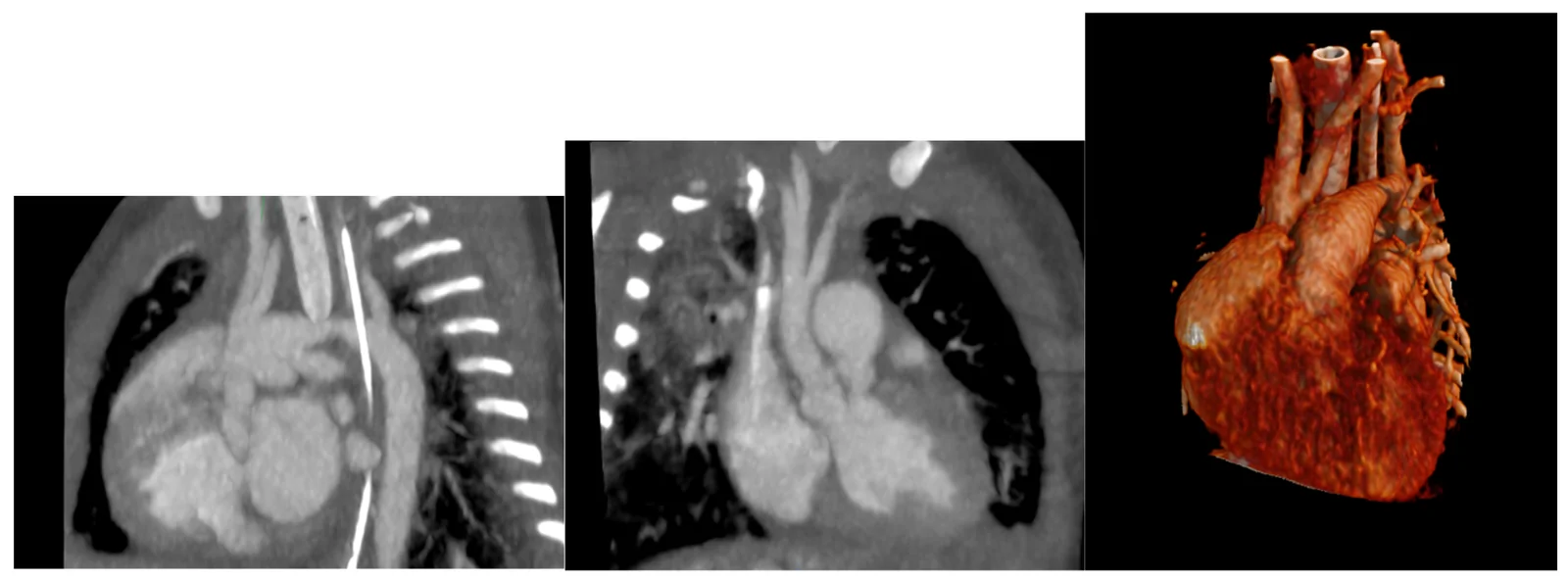
A patient with a type B interrupted aortic arch.

**Figure 5:**
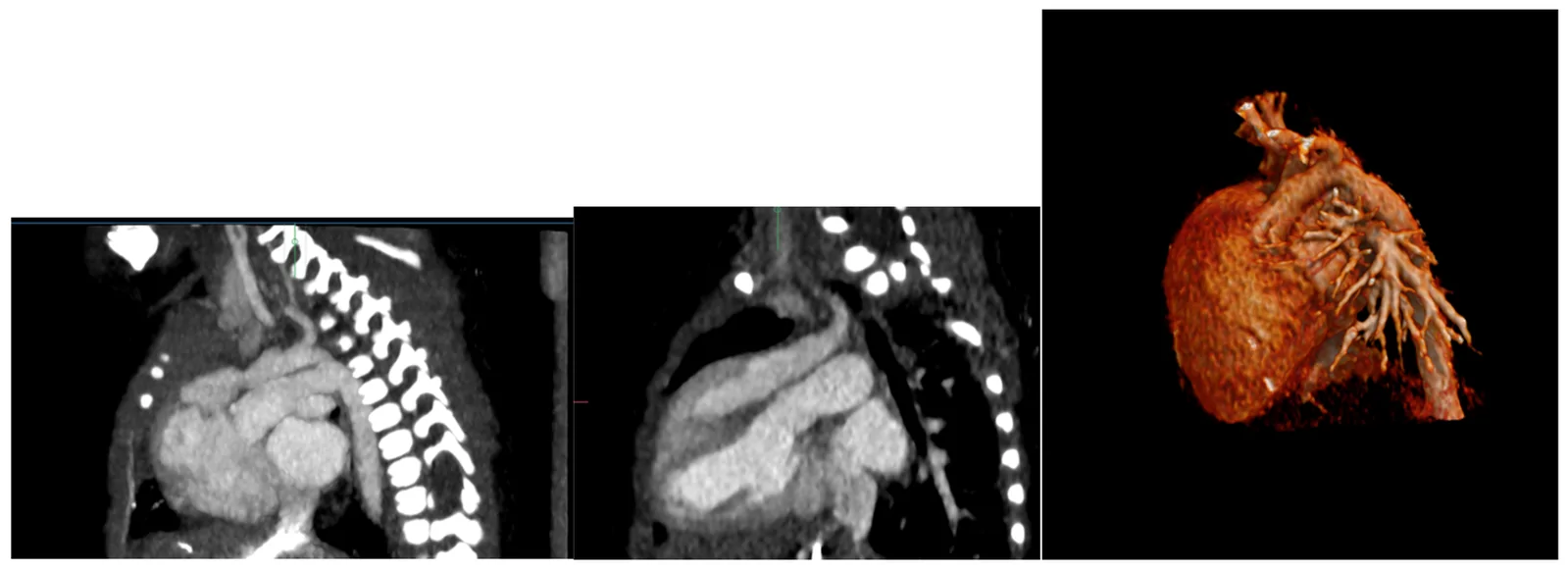
A patient with aortic arch and isthmus hypoplasia.

**Table 1:** Baseline demographic, anthropometric, and clinical characteristics of patients with conotruncal anomalies undergoing both cardiac CT angiography and echocardiography (*n* = 239).

Variable	Value
**Demographics**	
Sex—male	150 (62.8%)
Sex—female	89 (37.2%)
Age at CTA, days—median (IQR)	120 (4–196)
Neonate (≤28 days)	101 (42.3%)
Infant (29–365 days)	114 (47.7%)
Child (1–5 years)	19 (7.9%)
Child >5 years	5 (2.1%)
Interval from CTA to surgery, days—median (IQR)	7 (4–13)
Interval from TTE to CTA, days—median (IQR)	1 (1–2)
Body weight, kg—median (IQR)	5.3 (3.1–7.4)
BSA, m^2^—median (IQR)	0.30 (0.21–0.37)
Weight percentile—median (IQR)	20.1 (3.7–54.3)
Weight SDS—median (IQR)	−0.84 (−1.79–0.11)
Height, cm—median (IQR)	60.0 (49.0–66.0)
Height percentile—median (IQR)	18.2 (7.3–51.8)
Height SDS—median (IQR)	−0.91 (−1.45–0.05)
BMI, kg·m^−2^—median (IQR)	14.2 (12.8–16.6)
BMI percentile—median (IQR)	28.6 (3.5–67.3)
BMI SDS—median (IQR)	−0.56 (−1.82–0.45)
**Imaging parameters**	
Oxygen saturation, %—median (IQR)	89 (83–95)
Contrast volume, cc—median (IQR)	6 (5–9)
Contrast volume, ml·kg^−1^—median (IQR)	1.4 (1.2–1.6)
Effective radiation dose, mSv—median (IQR)	2.18 (1.79–2.82)
**Diagnosis**	
Tetralogy of Fallot (TOF)	94 (39.3%)
Double-outlet right ventricle (DORV)	50 (20.9%)
Transposition of the great arteries (TGA)	44 (18.4%)
Pulmonary atresia with VSD (PA-VSD)	26 (10.9%)
Truncus arteriosus	9 (3.8%)
Aortopulmonary window (APW)	8 (3.3%)
Interrupted aortic arch (IAA)	8 (3.3%)

IQR, interquartile range; CTA, computed tomography angiography; BSA, body surface area; SDS, standard deviation score (z-score); BMI, body mass index; VSD, ventricular septal defect. Continuous variables are reported as median (IQR) unless otherwise stated. Weight, height, and BMI percentiles and SDS were calculated using the WHO Child Growth Standards for children ≤5 years and the CDC 2000 growth reference for children >5 years (sex- and age-specific, LMS method). BSA was computed using the Mosteller formula.

**Table 2:** Additional anatomical information provided by CT angiography over echocardiography, stratified by conotruncal diagnosis.

Diagnostic Group	*n*	No Value (0) *n* (%)	Minor (1) *n* (%)	Major (2) *n* (%)
All patients	239	7 (2.9%)	108 (45.2%)	124 (51.9%)
Tetralogy of Fallot (TOF)	94	1 (1.1%)	53 (56.4%)	40 (42.6%)
Double-outlet right ventricle (DORV)	50	2 (4.0%)	22 (44.0%)	26 (52.0%)
Transposition of great arteries (TGA)	44	3 (6.8%)	19 (43.2%)	22 (50.0%)
Pulmonary atresia with VSD (PA-VSD)	26	0 (0.0%)	6 (23.1%)	20 (76.9%)
Truncus arteriosus	9	1 (11.1%)	3 (33.3%)	5 (55.6%)
Aortopulmonary window (APW)	8	0 (0.0%)	2 (25.0%)	6 (75.0%)
Interrupted aortic arch (IAA)	8	0 (0.0%)	3 (37.5%)	5 (62.5%)

CTA, computed tomography angiography; VSD, ventricular septal defect. The additional anatomical information provided by CTA over TTE was scored as 0 (no additional information), 1 (minor), or 2 (major) by consensus of two reviewers. Major additional anatomical information was frequent across all diagnostic subgroups (range 42.6–76.9%), without a statistically significant difference between groups (Freeman–Halton extension of Fisher’s exact test, *p* = 0.057), supporting a consistent additional anatomical contribution of CTA across the conotruncal anomaly spectrum.

**Table 3:** Diagnostic performance of TTE and CTA against surgical findings, with 95% confidence intervals and likelihood ratios (*n* = 239).

Domain	Modality	Sensitivity % (95% CI)	Specificity % (95% CI)	PPV %	NPV %	Accuracy %	LR+	LR−
Coronary anomaly	TTE	50.0 (37–63)	90.8 (86–94)	61.4	86.2	81.6	5.4	0.55
	**CTA**	**91.7 (82–96)**	**99.4 (97–100)**	**98.2**	**97.3**	**97.5**	**164.1**	**0.08**
Right aortic arch	TTE	87.5 (76–94)	98.9 (96–100)	96.1	96.3	96.2	80.1	0.13
	**CTA**	**96.4 (88–99)**	**98.4 (95–99)**	**94.7**	**98.9**	**97.9**	**58.8**	**0.04**
Aortic arch hypoplasia	TTE	87.5 (69–96)	98.1 (95–99)	84.0	98.6	97.1	47.0	0.13
	**CTA**	**100.0 (86–100)**	**100.0 (98–100)**	**100.0**	**100.0**	**100.0**	**NC**	**0.00**
Interrupted aortic arch	TTE	88.9 (67–97)	99.1 (97–100)	88.9	99.1	98.3	98.2	0.11
	**CTA**	**100.0 (82–100)**	**100.0 (98–100)**	**100.0**	**100.0**	**100.0**	**NC**	**0.00**
PA atresia	TTE	92.0 (75–98)	99.1 (97–100)	92.0	99.1	98.3	98.4	0.08
	**CTA**	**96.0 (80–99)**	**99.5 (97–100)**	**96.0**	**99.5**	**99.2**	**205.4**	**0.04**
PA non-confluence	TTE	50.0 (22–78)	99.6 (98–100)	80.0	98.3	97.9	115.5	0.50
	**CTA**	**87.5 (53–98)**	**100.0 (98–100)**	**100.0**	**99.6**	**99.6**	**NC**	**0.12**
PA hypoplasia	TTE	89.3 (82–94)	88.2 (81–93)	87.0	90.3	88.7	7.6	0.12
	**CTA**	**97.3 (92–99)**	**100.0 (97–100)**	**100.0**	**97.7**	**98.7**	**NC**	**0.03**

CI, confidence interval; CTA, computed tomography angiography; LR+, positive likelihood ratio; LR−, negative likelihood ratio; NC, not calculable; NPV, negative predictive value; PPV, positive predictive value; TTE, transthoracic echocardiography. LR+ was not calculable (NC) when specificity was 100% (no false positives). Coronary artery analysis was based on 233 patients after exclusion of 6 patients with indeterminate echocardiographic coronary assessment.

**Table 4:** Paired comparison of TTE and CTA against surgical findings (McNemar test).

Domain	Both Correct	TTE Only	CTA Only	Both İncorrect	McNemar *p*
Coronary anomaly	187	2	40	4	<0.001
Right aortic arch	226	4	8	1	0.388
Aortic arch hypoplasia	232	0	7	0	0.016
Interrupted aortic arch	235	0	4	0	0.125
PA atresia	234	1	3	1	0.625
PA non-confluence	234	0	4	1	0.125
PA hypoplasia	210	2	26	1	<0.001

“TTE only” = correctly classified by TTE but not CTA; “CTA only” = correctly classified by CTA but not TTE, relative to surgical findings. CTA, computed tomography angiography; TTE, transthoracic echocardiography. The coronary anomaly comparison was based on 233 patients after exclusion of 6 with indeterminate echocardiographic coronary assessment; all other domains were analyzed in the full cohort (*n* = 239).

## Data Availability

The data presented in this study are available on request from the corresponding author upon reasonable request. The data are not publicly available due to privacy and ethical restrictions.

## Supplementary Materials

The following supporting information can be downloaded at: https://www.mdpi.com/article/10.3390/diagnostics16193105/s1, Table S1: Inter-rater reliability of the CTA incremental contribution score.
