# Supplementary material for: Incremental Anatomical Value of CT Angiography Beyond Echocardiography in the Preoperative Assessment of Pediatric Conotruncal Anomalies: A Surgical Correlation Study
Source: Diagnostics (Basel). 2026 Sep 24;16(19):3105. doi: 10.3390/diagnostics16193105 (PMC13663931; doi:10.3390/diagnostics16193105)
Supplement: Supplementary file 1 [file diagnostics-16-03105-s001.zip › diagnostics-4476080-supplementary.pdf]

### Supplementary Table S1. Inter-rater reliability of the CTA incremental contribution score

Two reviewers independently scored the incremental contribution of CTA (0 = none, 1 = minor, 2 = major) in all 239 patients. Raw agreement was 89.5% (214/239); all 25 discordant cases differed by a single adjacent category, with no two-category (0 vs 2) disagreements. Discordant cases were resolved by consensus.

| **Agreement measure** | **κ** | **95% CI (bootstrap)** |
|---|---|---|
| Unweighted Cohen's κ | 0.797 | 0.722–0.862 |
| Linear weighted κ | 0.808 | 0.739–0.868 |
| **Quadratic weighted κ** | **0.825** | **0.763–0.881** |

*κ, Cohen's kappa; CI, confidence interval (2000 bootstrap resamples). Quadratic weighted κ = 0.825 (95% CI 0.763–0.881) indicates almost perfect agreement.*
